# 1-(4-Fluoro­phen­yl)-3-methyl-4-phenyl­sulfanyl-1*H*-pyrazol-5(4*H*)-one

**DOI:** 10.1107/S1600536810040596

**Published:** 2010-10-20

**Authors:** Tara Shahani, Hoong-Kun Fun, R. Venkat Ragavan, V. Vijayakumar, M. Venkatesh

**Affiliations:** a X-ray Crystallography Unit, School of Physics, Universiti Sains Malaysia, 11800 USM, Penang, Malaysia; b Organic Chemistry Division, School of Advanced Sciences, VIT University, Vellore-632 014, India; c Organic Chemistry Division, School of Advanced Sciences, VIT University, Vellore 632 014, India

## Abstract

The title compound, C_16_H_13_FN_2_OS, has undergone enol-to-keto tautomerism during the crystallization process. The 1*H*-pyrazole-5-one ring [maximum deviation = 0.0198 (11) Å] is inclined at angles of 33.10 (5) and 79.57 (5)° with respect to the fluoro­phenyl [maximum deviation = 0.0090 (12) Å] and phenyl­thiol [maximum deviation = 0.0229 (3) Å] rings attached to it. In the crystal, neighbouring mol­ecules are linked into inversion dimers, generating *R*_2_^2^(8) ring motifs. These dimers are further linked into two-dimensional arrays parallel to the *bc* plane *via* inter­molecular N—H⋯O, C—H⋯F and C—H⋯O hydrogen bonds. The crystal is further stabilized by weak π–π [centroid–centroid distance = 3.6921 (7) Å] and C—H⋯π inter­actions.

## Related literature

For pyrazole derivatives and their microbial activity, see: Ragavan *et al.* (2009 ▶, 2010 ▶). For related structures, see: Shahani *et al.* (2009 ▶, 2010*a* ▶,*b* ▶,*c* ▶). For hydrogen-bond motifs, see: Bernstein *et al.* (1995 ▶). For bond-length data, see: Allen *et al.* (1987 ▶). For the stability of the temperature controller used in the data collection, see: Cosier & Glazer (1986 ▶).
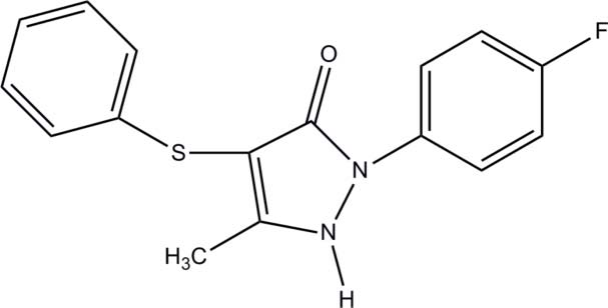


## Experimental

### 

#### Crystal data

C_16_H_13_FN_2_OS
*M* *_r_* = 300.34
Monoclinic, 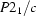

*a* = 17.2628 (3) Å
*b* = 7.28340 (1) Å
*c* = 11.4877 (2) Å
β = 91.138 (1)°
*V* = 1444.09 (4) Å^3^
*Z* = 4
Mo *K*α radiation
μ = 0.24 mm^−1^
*T* = 100 K
0.37 × 0.17 × 0.14 mm

#### Data collection

Bruker SMART APEXII CCD diffractometer
Absorption correction: multi-scan (*SADABS*; Bruker, 2009 ▶) *T*_min_ = 0.918, *T*_max_ = 0.968
21517 measured reflections
5704 independent reflections
4543 reflections with *I* > 2σ(*I*)
*R*_int_ = 0.037

#### Refinement

*R*[*F* ^2^ > 2σ(*F* ^2^)] = 0.043
*wR*(*F* ^2^) = 0.113
*S* = 1.03
5704 reflections
195 parameters
H atoms treated by a mixture of independent and constrained refinement
Δρ_max_ = 0.48 e Å^−3^
Δρ_min_ = −0.28 e Å^−3^

### 

Data collection: *APEX2* (Bruker, 2009 ▶); cell refinement: *SAINT* (Bruker, 2009 ▶); data reduction: *SAINT*; program(s) used to solve structure: *SHELXTL* (Sheldrick, 2008 ▶); program(s) used to refine structure: *SHELXTL*; molecular graphics: *SHELXTL*; software used to prepare material for publication: *SHELXTL* and *PLATON* (Spek, 2009 ▶).

## Supplementary Material

Crystal structure: contains datablocks global, I. DOI: 10.1107/S1600536810040596/hb5673sup1.cif

Structure factors: contains datablocks I. DOI: 10.1107/S1600536810040596/hb5673Isup2.hkl

Additional supplementary materials:  crystallographic information; 3D view; checkCIF report

## Figures and Tables

**Table 1:** Hydrogen-bond geometry (Å, °) *Cg*1 and *Cg*3 are the centroids of the pyrazol (N1/N2/C7–C9) and benzene (C10–C15) rings, respectively.

*D*—H⋯*A*	*D*—H	H⋯*A*	*D*⋯*A*	*D*—H⋯*A*
N2—H1*N*2⋯O1^i^	0.93 (2)	1.72 (2)	2.6352 (12)	168 (2)
C2—H2*A*⋯F1^ii^	0.93	2.49	3.1450 (16)	128
C4—H4*A*⋯F1^iii^	0.93	2.43	3.2381 (15)	145
C5—H5*A*⋯O1^i^	0.93	2.56	3.2786 (15)	134
C2—H2*A*⋯*Cg*1^iv^	0.93	2.94	3.6300 (14)	132
C12—H12*A*⋯*Cg*3^v^	0.93	2.74	3.5928 (14)	153
C16—H16*B*⋯*Cg*3^vi^	0.96	2.79	3.6826 (13)	155

Symmetry codes: (i) 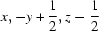; (ii) 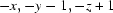; (iii) 
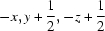
; (iv) 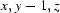; (v) 
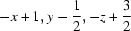
; (vi) 
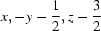
.

**Fig. 1.**
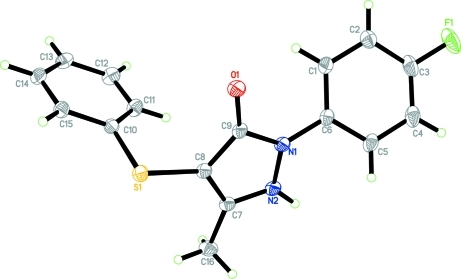
The molecular structure of the title compound, showing 50% probability displacement ellipsoids.

**Fig. 2.**
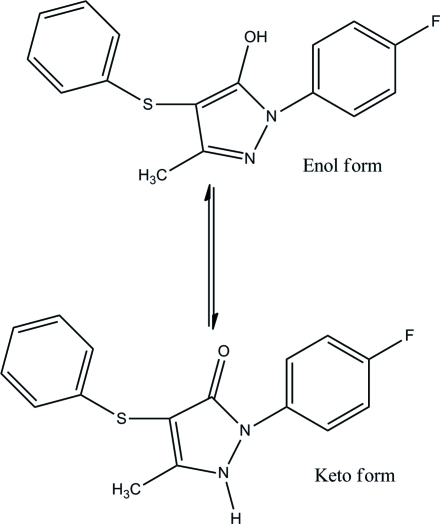
Enol-to-keto tautomerism of the title compound during crystallization process.

**Fig. 3.**
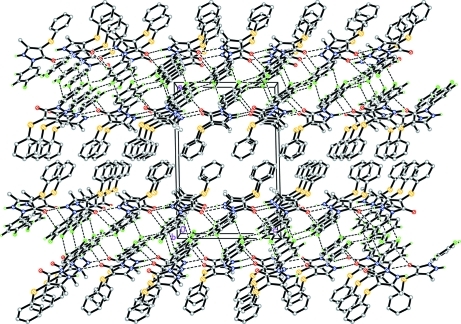
The crystal packing of the title compound, showing two two-dimensional arrays parallel to the bc plane. Intermolecular hydrogen bonds are shown as dashed lines.

## supplementary crystallographic information

### Comment

Antibacterial and antifungal activities of the azoles are most widely studied and some of them are in clinical practice as anti-microbial agents. However, the azole-resistant strain had led to the development of new antimicrobial compounds. In particular pyrazole derivatives are extensively studied and used as antimicrobial agents. Pyrazole is an important class of heterocyclic compounds and many pyrazole derivatives are reported to have the broad spectrum of biological properties, such as anti-inflammatory, antifungal, herbicidal, anti-tumour, cytotoxic, molecular modelling, and antiviral activities. Pyrazole derivatives also act as antiangiogenic agents, A3 adenosine receptor antagonists, neuropeptide YY5 receptor antagonists, kinase inhibitor for treatment of type 2 diabetes, hyperlipidemia, obesity, and thrombopiotinmimetics. Recently urea derivatives of pyrazoles have been reported as potent inhibitors of p38 kinase. Since the high electronegativity of halogens (particularly chlorine and fluorine) in the aromatic part of the drug molecules play an important role in enhancing their biological activity, we are interested to have 4-fluoro or 4-chloro substitution in the aryls of 1,5-diaryl pyrazoles. As part of our on-going research aiming the synthesis of new antimicrobial compounds, we have reported the synthesis of novel pyrazole derivatives and their microbial activities (Ragavan *et al.*, 2009; 2010). The structure of the title compound is presented here. The synthesis lead to the enol form of the compound (see Ragavan *et al.*, 2009). However the single crystal structure determination gives the keto form. Therefore the compound undergoes an enol-to-keto tautomerism during crystallization. The interconversion of the two forms involves the movement of a proton and the shifting of bonding electrons; hence, the isomerism qualifies as tautomerism (Fig. 2)

The asymmetric unit of the title compound, (Fig. 1), consists of three rings, namely fluorophenyl (F1/C1–C6), 5-3methyl-2,5dihydro-1*H*-pyrazol-3-one (N1/N2/C7–C9/O1/C16) and phenylthiol (S1/C10–C15).The 1-(4-fluorophenyl)-3-methyl-4-(phenylthio)-1*H*-pyrazol-5-ol undergoes an enol-to-ketotautomerism during the crystallization process (Fig. 2). The 1*H*-pyrazole-5-one ring (maximum deviation 0.0198 (11) Å at atom C8) is inclined at angles of 33.10 (5) and 79.57 (5)° with respect to the fluorophenyl (maximum deviation 0.0090 (12) at atom C2) and phenylthiol (maximum deviation 0.0229 (3) at atom S1) rings attached to it. The bond lengths (Allen *et al.*, 1987) and angles are within normal ranges and comparable to the closely related structures (Shahani *et al.*, 2009; 2010*a*,*b*).

In the crystal packing (Fig. 3), intermolecular C2—H2A···F1 hydrogen bonds (Table 1) link the neighbouring molecules into dimers, generating *R*^2^_2_(8) ring motifs (Bernstein *et al.*, 1995). These dimers are further linked into two-dimensional arrays parallel to the *bc* plane by intermolecular N2—H1N2···O1, C2—H2A···F1, C4—H4A···F1 and C5—H5A···O1 hydrogen bonds (Table 1). Weak π–π interactions were observed [*Cg*2···*Cg*2 = 3.6921 (7) Å, symmetry code = –*X*, –Y, 1-*Z*], *Cg*2 is the centroid of the benzene ring (C1–C6). The crystal structure is further stabilized by C—H···π interactions (Table 1), involving the C10–C15 (centroid *Cg* 1) and N1/N2/C7/C8/C9 rings (centroid *Cg*3).

### Experimental

The compound has been synthesized using the method available in the literature (Ragavan *et al.*, 2009) and recrystallized using an ethanol-chloroform 1:1 mixture to generate colourless needles of (I). Yield: 58%. M.Pt: 475 K.

### Refinement

The hydrogen atoms bound to C atoms were positioned geometrically [C–H = 0.9300 to 0.9600 Å] with *U*_iso_(H) =1.2 or 1.5*U*_iso_(C). The hydrogen atom attached to the N2 atom was located from the difference map and refined freely.

### Crystal data

**Table:**

C_16_H_13_FN_2_OS	*F*(000) = 624
*M**_r_* = 300.34	*D*_x_ = 1.381 Mg m^−^^3^
Monoclinic, *P*2_1_/*c*	Mo *K*α radiation, λ = 0.71073 Å
Hall symbol: -P 2ybc	Cell parameters from 5911 reflections
*a* = 17.2628 (3) Å	θ = 2.4–33.6°
*b* = 7.28340 (1) Å	µ = 0.24 mm^−^^1^
*c* = 11.4877 (2) Å	*T* = 100 K
β = 91.138 (1)°	Needle, colourless
*V* = 1444.09 (4) Å^3^	0.37 × 0.17 × 0.14 mm
*Z* = 4	

### Data collection

**Table:**

Bruker SMART APEXII CCD diffractometer	5704 independent reflections
Radiation source: fine-focus sealed tube	4543 reflections with *I* > 2σ(*I*)
graphite	*R*_int_ = 0.037
φ and ω scans	θ_max_ = 33.6°, θ_min_ = 2.4°
Absorption correction: multi-scan (*SADABS*; Bruker, 2009)	*h* = −25→26
*T*_min_ = 0.918, *T*_max_ = 0.968	*k* = −9→11
21517 measured reflections	*l* = −17→17

### Refinement

**Table:**

Refinement on *F*^2^	Primary atom site location: structure-invariant direct methods
Least-squares matrix: full	Secondary atom site location: difference Fourier map
*R*[*F*^2^ > 2σ(*F*^2^)] = 0.043	Hydrogen site location: inferred from neighbouring sites
*wR*(*F*^2^) = 0.113	H atoms treated by a mixture of independent and constrained refinement
*S* = 1.03	*w* = 1/[σ^2^(*F*_o_^2^) + (0.0502*P*)^2^ + 0.527*P*] where *P* = (*F*_o_^2^ + 2*F*_c_^2^)/3
5704 reflections	(Δ/σ)_max_ < 0.001
195 parameters	Δρ_max_ = 0.48 e Å^−^^3^
0 restraints	Δρ_min_ = −0.28 e Å^−^^3^

### Special details

**Table:**

Experimental. The crystal was placed in the cold stream of an Oxford Cyrosystems Cobra open-flow nitrogen cryostat (Cosier & Glazer, 1986) operating at 100.0 (1) K.
Geometry. All e.s.d.'s (except the e.s.d. in the dihedral angle between two l.s. planes) are estimated using the full covariance matrix. The cell e.s.d.'s are taken into account individually in the estimation of e.s.d.'s in distances, angles and torsion angles; correlations between e.s.d.'s in cell parameters are only used when they are defined by crystal symmetry. An approximate (isotropic) treatment of cell e.s.d.'s is used for estimating e.s.d.'s involving l.s. planes.
Refinement. Refinement of *F*^2^ against ALL reflections. The weighted *R*-factor *wR* and goodness of fit *S* are based on *F*^2^, conventional *R*-factors *R* are based on *F*, with *F* set to zero for negative *F*^2^. The threshold expression of *F*^2^ > σ(*F*^2^) is used only for calculating *R*-factors(gt) *etc*. and is not relevant to the choice of reflections for refinement. *R*-factors based on *F*^2^ are statistically about twice as large as those based on *F*, and *R*- factors based on ALL data will be even larger.

### Fractional atomic coordinates and isotropic or equivalent isotropic displacement parameters (Å^2^)

**Table:**

	*x*	*y*	*z*	*U*_iso_*/*U*_eq_	
S1	0.287633 (16)	0.50655 (4)	0.76602 (2)	0.01812 (7)	
F1	0.01268 (6)	−0.41073 (14)	0.38015 (8)	0.0433 (3)	
O1	0.17691 (5)	0.14246 (12)	0.74551 (7)	0.02106 (17)	
N1	0.17513 (6)	0.18952 (13)	0.54527 (8)	0.01725 (18)	
N2	0.20721 (6)	0.31449 (14)	0.46958 (8)	0.01727 (18)	
C1	0.13682 (7)	−0.12951 (16)	0.56378 (10)	0.0193 (2)	
H1A	0.1672	−0.1386	0.6313	0.023*	
C2	0.09597 (7)	−0.28107 (18)	0.52250 (11)	0.0231 (2)	
H2A	0.0978	−0.3924	0.5621	0.028*	
C3	0.05246 (8)	−0.2620 (2)	0.42099 (11)	0.0271 (3)	
C4	0.04640 (7)	−0.1000 (2)	0.36073 (10)	0.0285 (3)	
H4A	0.0161	−0.0924	0.2930	0.034*	
C5	0.08635 (7)	0.05268 (19)	0.40275 (10)	0.0226 (2)	
H5A	0.0826	0.1646	0.3641	0.027*	
C6	0.13208 (6)	0.03598 (16)	0.50366 (9)	0.0166 (2)	
C7	0.24940 (6)	0.43573 (15)	0.53067 (9)	0.01692 (19)	
C8	0.24443 (6)	0.39363 (15)	0.64842 (9)	0.01656 (19)	
C9	0.19702 (6)	0.23394 (15)	0.65843 (9)	0.01662 (19)	
C10	0.36501 (6)	0.35655 (15)	0.80605 (9)	0.01676 (19)	
C11	0.39018 (7)	0.21470 (17)	0.73533 (10)	0.0211 (2)	
H11A	0.3652	0.1923	0.6643	0.025*	
C12	0.45278 (7)	0.10600 (18)	0.77057 (11)	0.0238 (2)	
H12A	0.4694	0.0114	0.7228	0.029*	
C13	0.49050 (7)	0.13786 (18)	0.87642 (11)	0.0231 (2)	
H13A	0.5329	0.0666	0.8991	0.028*	
C14	0.46437 (7)	0.27724 (17)	0.94827 (10)	0.0220 (2)	
H14A	0.4890	0.2980	1.0198	0.026*	
C15	0.40165 (7)	0.38605 (17)	0.91402 (10)	0.0199 (2)	
H15A	0.3842	0.4783	0.9629	0.024*	
C16	0.29242 (7)	0.58417 (17)	0.47078 (10)	0.0228 (2)	
H16A	0.2592	0.6404	0.4130	0.034*	
H16B	0.3370	0.5331	0.4340	0.034*	
H16C	0.3088	0.6748	0.5267	0.034*	
H1N2	0.1992 (11)	0.314 (3)	0.3895 (18)	0.049 (6)*	

### Atomic displacement parameters (Å^2^)

**Table:**

	*U*^11^	*U*^22^	*U*^33^	*U*^12^	*U*^13^	*U*^23^
S1	0.02393 (14)	0.01636 (13)	0.01404 (12)	0.00137 (10)	−0.00028 (9)	−0.00369 (9)
F1	0.0523 (6)	0.0484 (6)	0.0292 (4)	−0.0311 (5)	−0.0029 (4)	−0.0083 (4)
O1	0.0323 (4)	0.0221 (4)	0.0089 (3)	−0.0046 (3)	0.0013 (3)	0.0002 (3)
N1	0.0241 (4)	0.0185 (4)	0.0092 (4)	−0.0017 (3)	0.0003 (3)	0.0006 (3)
N2	0.0237 (4)	0.0192 (4)	0.0090 (4)	0.0002 (3)	0.0013 (3)	0.0012 (3)
C1	0.0193 (5)	0.0206 (5)	0.0179 (5)	0.0009 (4)	−0.0019 (4)	−0.0015 (4)
C2	0.0234 (5)	0.0226 (5)	0.0232 (5)	−0.0027 (4)	0.0001 (4)	−0.0022 (4)
C3	0.0268 (6)	0.0346 (7)	0.0200 (5)	−0.0133 (5)	0.0019 (4)	−0.0074 (5)
C4	0.0260 (6)	0.0451 (8)	0.0143 (5)	−0.0124 (5)	−0.0026 (4)	0.0009 (5)
C5	0.0215 (5)	0.0331 (6)	0.0132 (5)	−0.0032 (5)	−0.0020 (4)	0.0028 (4)
C6	0.0168 (4)	0.0212 (5)	0.0118 (4)	0.0002 (4)	0.0007 (3)	−0.0026 (4)
C7	0.0212 (5)	0.0166 (5)	0.0130 (4)	0.0022 (4)	0.0018 (3)	0.0002 (4)
C8	0.0221 (5)	0.0163 (5)	0.0114 (4)	0.0009 (4)	0.0009 (3)	−0.0011 (4)
C9	0.0225 (5)	0.0182 (5)	0.0092 (4)	0.0009 (4)	−0.0002 (3)	−0.0015 (3)
C10	0.0198 (5)	0.0173 (5)	0.0131 (4)	−0.0018 (4)	0.0015 (3)	0.0000 (4)
C11	0.0246 (5)	0.0244 (5)	0.0144 (5)	0.0027 (4)	0.0011 (4)	−0.0037 (4)
C12	0.0273 (6)	0.0255 (6)	0.0189 (5)	0.0056 (5)	0.0037 (4)	−0.0023 (4)
C13	0.0227 (5)	0.0271 (6)	0.0196 (5)	0.0021 (4)	0.0018 (4)	0.0051 (4)
C14	0.0245 (5)	0.0257 (6)	0.0158 (5)	−0.0033 (4)	−0.0015 (4)	0.0031 (4)
C15	0.0252 (5)	0.0210 (5)	0.0134 (4)	−0.0027 (4)	0.0008 (4)	−0.0013 (4)
C16	0.0295 (6)	0.0205 (5)	0.0186 (5)	−0.0013 (4)	0.0052 (4)	0.0030 (4)

### Geometric parameters (Å, °)

**Table:**

S1—C8	1.7371 (11)	C5—H5A	0.9300
S1—C10	1.7790 (11)	C7—C8	1.3913 (15)
F1—C3	1.3613 (15)	C7—C16	1.4879 (17)
O1—C9	1.2567 (13)	C8—C9	1.4280 (16)
N1—N2	1.3821 (13)	C10—C11	1.3894 (16)
N1—C9	1.3848 (13)	C10—C15	1.3977 (15)
N1—C6	1.4203 (14)	C11—C12	1.3933 (17)
N2—C7	1.3351 (14)	C11—H11A	0.9300
N2—H1N2	0.93 (2)	C12—C13	1.3873 (17)
C1—C2	1.3884 (16)	C12—H12A	0.9300
C1—C6	1.3908 (16)	C13—C14	1.3893 (18)
C1—H1A	0.9300	C13—H13A	0.9300
C2—C3	1.3814 (17)	C14—C15	1.3922 (17)
C2—H2A	0.9300	C14—H14A	0.9300
C3—C4	1.371 (2)	C15—H15A	0.9300
C4—C5	1.3898 (18)	C16—H16A	0.9600
C4—H4A	0.9300	C16—H16B	0.9600
C5—C6	1.3948 (15)	C16—H16C	0.9600
			
C8—S1—C10	102.64 (5)	C7—C8—S1	128.16 (9)
N2—N1—C9	109.36 (9)	C9—C8—S1	124.12 (8)
N2—N1—C6	121.35 (8)	O1—C9—N1	123.29 (10)
C9—N1—C6	129.13 (9)	O1—C9—C8	131.59 (10)
C7—N2—N1	109.03 (9)	N1—C9—C8	105.12 (9)
C7—N2—H1N2	126.2 (13)	C11—C10—C15	119.47 (11)
N1—N2—H1N2	124.7 (13)	C11—C10—S1	123.19 (8)
C2—C1—C6	119.67 (10)	C15—C10—S1	117.34 (9)
C2—C1—H1A	120.2	C10—C11—C12	120.16 (10)
C6—C1—H1A	120.2	C10—C11—H11A	119.9
C3—C2—C1	118.17 (12)	C12—C11—H11A	119.9
C3—C2—H2A	120.9	C13—C12—C11	120.50 (11)
C1—C2—H2A	120.9	C13—C12—H12A	119.8
F1—C3—C4	118.56 (11)	C11—C12—H12A	119.8
F1—C3—C2	118.22 (13)	C12—C13—C14	119.39 (11)
C4—C3—C2	123.22 (12)	C12—C13—H13A	120.3
C3—C4—C5	118.73 (11)	C14—C13—H13A	120.3
C3—C4—H4A	120.6	C13—C14—C15	120.52 (10)
C5—C4—H4A	120.6	C13—C14—H14A	119.7
C4—C5—C6	119.19 (12)	C15—C14—H14A	119.7
C4—C5—H5A	120.4	C14—C15—C10	119.94 (11)
C6—C5—H5A	120.4	C14—C15—H15A	120.0
C1—C6—C5	121.00 (11)	C10—C15—H15A	120.0
C1—C6—N1	119.34 (9)	C7—C16—H16A	109.5
C5—C6—N1	119.66 (11)	C7—C16—H16B	109.5
N2—C7—C8	108.77 (10)	H16A—C16—H16B	109.5
N2—C7—C16	120.64 (10)	C7—C16—H16C	109.5
C8—C7—C16	130.59 (10)	H16A—C16—H16C	109.5
C7—C8—C9	107.72 (9)	H16B—C16—H16C	109.5
			
C9—N1—N2—C7	0.64 (12)	C16—C7—C8—S1	0.70 (19)
C6—N1—N2—C7	−175.10 (10)	C10—S1—C8—C7	−104.41 (11)
C6—C1—C2—C3	0.90 (18)	C10—S1—C8—C9	74.55 (10)
C1—C2—C3—F1	179.69 (12)	N2—N1—C9—O1	−179.24 (10)
C1—C2—C3—C4	−1.5 (2)	C6—N1—C9—O1	−3.94 (18)
F1—C3—C4—C5	179.41 (12)	N2—N1—C9—C8	0.04 (12)
C2—C3—C4—C5	0.6 (2)	C6—N1—C9—C8	175.35 (11)
C3—C4—C5—C6	0.9 (2)	C7—C8—C9—O1	178.53 (12)
C2—C1—C6—C5	0.50 (18)	S1—C8—C9—O1	−0.61 (19)
C2—C1—C6—N1	−178.88 (11)	C7—C8—C9—N1	−0.68 (12)
C4—C5—C6—C1	−1.40 (18)	S1—C8—C9—N1	−179.82 (8)
C4—C5—C6—N1	177.98 (11)	C8—S1—C10—C11	14.04 (11)
N2—N1—C6—C1	144.74 (11)	C8—S1—C10—C15	−166.31 (9)
C9—N1—C6—C1	−30.08 (17)	C15—C10—C11—C12	−1.69 (18)
N2—N1—C6—C5	−34.65 (16)	S1—C10—C11—C12	177.96 (10)
C9—N1—C6—C5	150.54 (12)	C10—C11—C12—C13	0.10 (19)
N1—N2—C7—C8	−1.07 (12)	C11—C12—C13—C14	1.21 (19)
N1—N2—C7—C16	178.48 (10)	C12—C13—C14—C15	−0.93 (19)
N2—C7—C8—C9	1.09 (13)	C13—C14—C15—C10	−0.66 (18)
C16—C7—C8—C9	−178.40 (11)	C11—C10—C15—C14	1.97 (17)
N2—C7—C8—S1	−179.82 (9)	S1—C10—C15—C14	−177.70 (9)

### Hydrogen-bond geometry (Å, °)

**Table:**

Cg1 and Cg3 are the centroids of the pyrazol (N1/N2/C7–C9) and benzene ring (C10–C15) rings, respectively.

**Table:**

*D*—H···*A*	*D*—H	H···*A*	*D*···*A*	*D*—H···*A*
N2—H1N2···O1^i^	0.93 (2)	1.72 (2)	2.6352 (12)	168 (2)
C2—H2A···F1^ii^	0.93	2.49	3.1450 (16)	128
C4—H4A···F1^iii^	0.93	2.43	3.2381 (15)	145
C5—H5A···O1^i^	0.93	2.56	3.2786 (15)	134
C2—H2A···Cg1^iv^	0.93	2.94	3.6300 (14)	132
C12—H12A···Cg3^v^	0.93	2.74	3.5928 (14)	153
C16—H16B···Cg3^vi^	0.96	2.79	3.6826 (13)	155

Symmetry codes: (i) *x*, −*y*+1/2, *z*−1/2; (ii) −*x*, −*y*−1, −*z*+1; (iii) −*x*, *y*+1/2, −*z*+1/2; (iv) *x*, *y*−1, *z*; (v) −*x*+1, *y*−1/2, −*z*+3/2; (vi) *x*, −*y*−1/2, *z*−3/2.
